# Two Cases of Malignant Melanoma with Long-term Survival after the Appearance of Brain Metastases

**DOI:** 10.31662/jmaj.2024-0400

**Published:** 2025-03-28

**Authors:** Kyosuke Oishi, Natsumi Fushida, Jiro Nishio, Ko Fujii, Motoki Horii, Kyoko Shimizu, Shintaro Maeda, Yasuhito Hamaguchi, Takashi Matsushita

**Affiliations:** 1 Department of Dermatology, Faculty of Medicine, Institute of Medical, Pharmaceutical and Health Sciences, Kanazawa University, Kanazawa, Japan

**Keywords:** malignant melanoma, brain metastases, long survival

## Abstract

Brain metastases from malignant tumors are generally known to have a poor prognosis. One of the major reasons for this is the lack of efficacy of anti-tumor drugs compared to other organs. One of the major reasons for this is the lack of efficacy of anti-tumor drugs compared to other organs. Malignant melanoma is a highly malignant tumor that occurs mainly in the skin and is relatively prone to brain metastasis. In this case report, we report two cases of malignant melanoma with brain metastases that were treated with a combination of radiotherapy and chemotherapy and had long-term survival. Case 1 was a 51-year-old Japanese man with primary melanoma of the chest; the pathological staging was pT4aN1aM0, stage IIIC. He developed multiple brain metastases two years and three months after the initial resection. Case 2 was a 23-year-old Japanese woman with primary melanoma of the upper extremities; the pathological staging was pT1bN1aM0, stage IIIA. She developed brain metastases one year and nine months after the initial resection. Both patients had positive BRAF gene mutations in their primary tumors. The combination of BRAF inhibitors, immune checkpoint inhibitors, and stereotactic radiotherapy resulted in long-term survival of more than 5 years for Case 1 and more than 6 years for Case 2. With advances in chemotherapy and radiotherapy, the prognosis for patients with brain metastases, not only malignant melanoma, is expected to improve further in the future. Although rare, malignant melanoma is known to occur in various organs other than the skin. We report this case because we believe that our case report will be of interest to physicians who treat the above organs.

## Introduction

Malignant melanoma is a highly malignant tumor, and the prognosis for patients with brain metastases is known to be quite poor. We report two cases of brain metastases from malignant melanoma that were treated with a combination of chemotherapy and stereotactic radiotherapy and achieved long-term survival.

## Case Report

### Case 1

**Figure 1.**
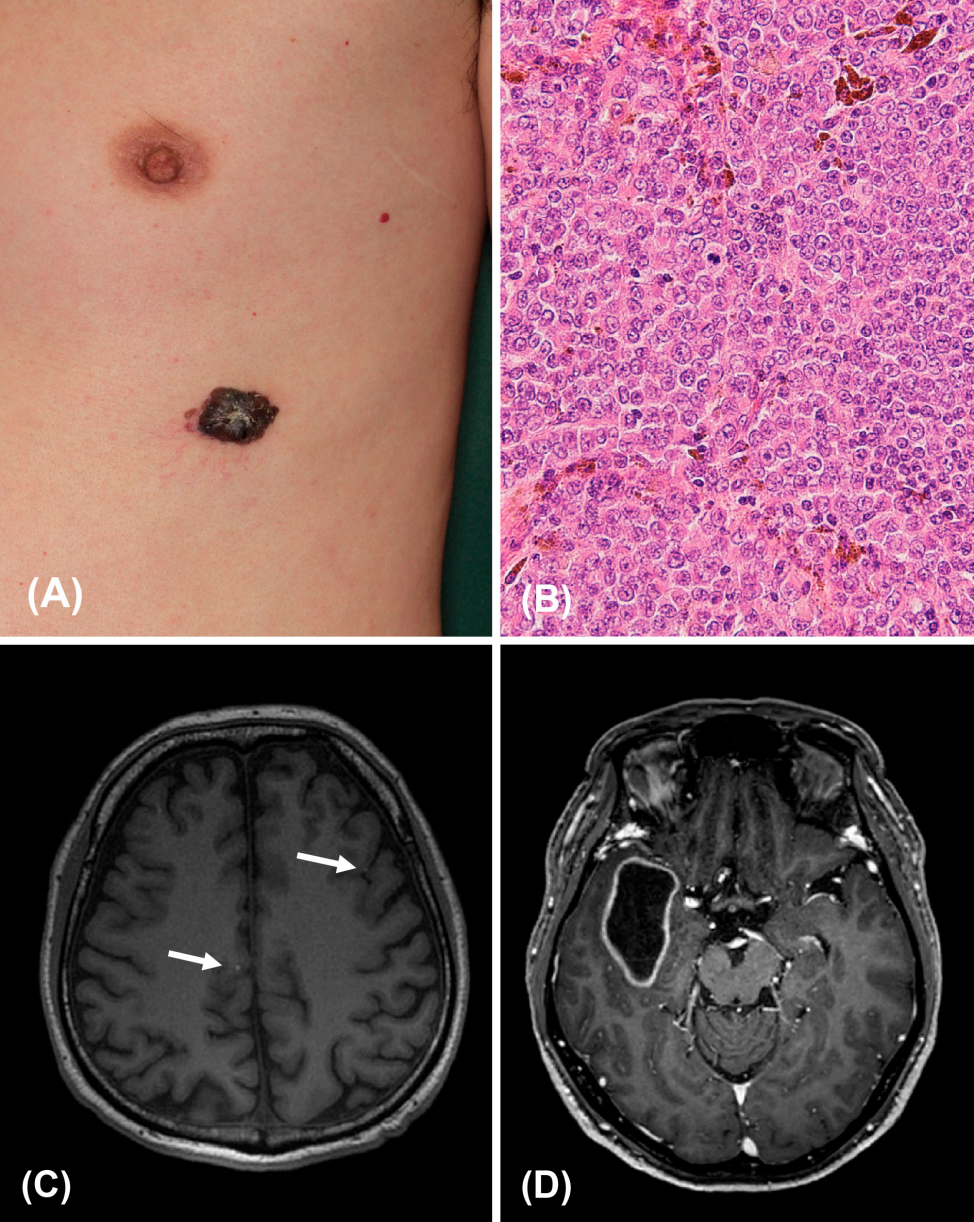
(A) Clinical photograph of Case 1. A black nodule on the left chest, with surrounding pigmented patches. (B) Pathological findings of Case 1. A dense proliferation of atypical melanocytes was seen in the lesion (Hematoxylin-eosin staining, original magnification, ×200). (C) Magnetic resonance imaging before the treatment with pembrolizumab and stereotactic radiotherapy in Case 1. Multiple brain metastases were seen (white arrows). (D) Magnetic resonance imaging 5 years after the treatment with pembrolizumab and stereotactic radiotherapy in Case 1.

A 51-year-old Japanese male had a pigmented spot on the left chest that had been present for 20 years, gradually increasing in size. A black nodule, 20 mm in diameter, was found on the left thoracic region, with surrounding pigmented patches (Figure 1A). The clinical diagnosis was malignant melanoma (MM). The patient underwent an extended resection and sentinel lymph node biopsy. The pathological diagnosis was MM (Figure 1B). The sentinel lymph node in the left axilla was positive for metastasis, and lymph node dissection was performed. The pathological staging was pT4aN1aM0, stage IIIC. Nine months after surgery, left lung metastasis appeared, and monotherapy with Dacarbazine started. Three months after the treatment, skin metastasis appeared, so DTIC was discontinued, and he began treatment with vemurafenib because his tumor was positive for BRAF mutations. Fourteen months after the start of vemurafenib therapy, magnetic resonance imaging revealed multiple brain metastases (BMs) (Figure 1C). After the appearance of BM, he switched from vemurafenib to pembrolizumab. Stereotactic radiotherapy (SRT) was also used when new BM appeared. BM gradually increased in number after starting pembrolizumab, and some formed cysts (Figure 1D). The disease progressively worsened, and the patient died of meningeal dissemination 5 years and 5 months after the appearance of BM.

### Case 2

**Figure 2.**
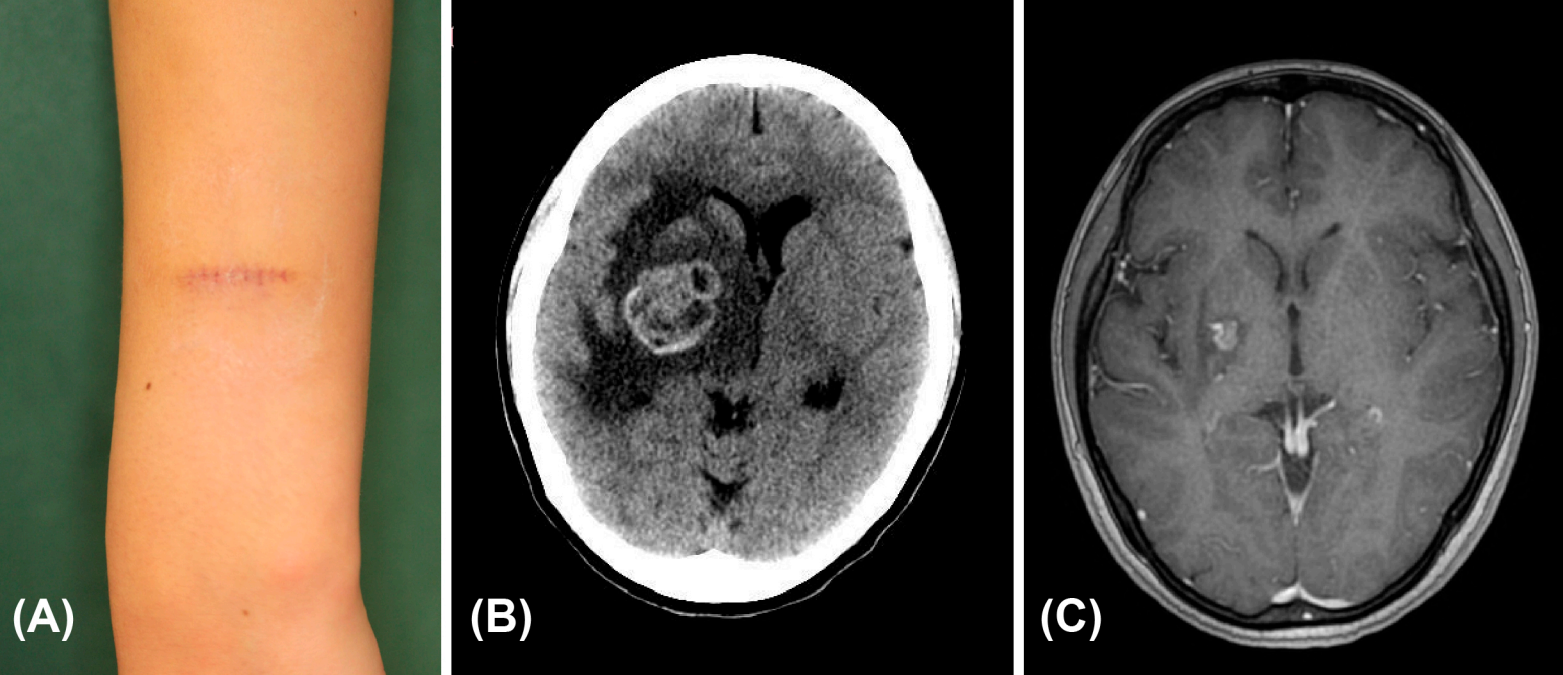
(A) Clinical photograph of Case 2. There were no residual lesions around the scar. (B) Computerized tomography before the treatment with chemotherapy and stereotactic radiotherapy in Case 2. Metastasis measuring 35 mm in diameter is seen in the right basal ganglia. (C) Magnetic resonance imaging 4 years after the treatment with pembrolizumab and stereotactic radiotherapy in Case 2. The size of metastasis has been significantly reduced.

A 23-year-old Japanese female had a pigmented spot on her left forearm since birth, which gradually increased in size 7 years ago. After excision at another hospital, she was diagnosed with MM and referred to our department. Clinical photographs taken at the time of the patient’s first visit to our hospital showed no residual lesions (Figure 2A). The sentinel lymph node in the left axilla was positive for metastasis, and lymph node dissection was performed. The pathological staging was pT1bN1aM0, stage IIIA. After 21 months from lymph node dissection, she developed headache and vomiting, and computerized tomography showed a BM measuring 35 mm in diameter (Figure 2B). Her tumor was positive for a BRAF mutation, but she could not take BRAF/MEK inhibitors due to symptoms originating from BM, so she initially received two courses of nivolumab infusion. Glucocorticoids and diuretics were administered to allow oral administration of medications, and dabrafenib and trametinib were started. She also received SRT in three separate sessions, which significantly reduced the size of her BM (Figure 2C). Six years and nine months have passed since the appearance of BM, and the patient continues to take dabrafenib and trametinib without any increase in BM or other metastatic lesions.

## Discussion

The response rate to chemotherapy for BM of MM is 44%-59% for BRAF/MEK inhibitors ^(1)^, 20% for anti-PD-1 antibody agents ^(2)^, 46% for combined anti-PD-1/anti-CTLA-4 antibody agents ^(2)^, and 36% for immune checkpoint inhibitors (ICIs) and radiation therapy ^(3)^. The γ-knife is one of the main instruments of SRT, and the local control rate for MM with BM is 86.2% at 12 months and 84% at 24 months ^(4)^. The median overall survival (OS) for MM with BM used to be several months, but recent reports indicate that it has been extended from 9 months to about 2 years due to ICI and BRAF/MEK inhibitors ^(5)^.

In a meta-analysis, OS was significantly better in patients who received ICI + SRT than in those who received SRT alone. However, this clinical benefit was not observed in patients who received BRAF/MEK inhibitors + SRT versus those who received SRT alone ^(6)^.

The reasons for the relatively good outcome of our cases are thought to be that Lactate Dehydrogenase was normal in both cases throughout the course, all distant metastases other than BM were resected in Case 1, and there were no distant metastases other than BM in Case 2. It has also been reported that ICI therapy and SRT performed in close proximity to each other improves prognosis ^(7)^, and it is possible that the treatment plan in our cases was designed that way, which may have had a positive impact.

Although rare, MM is known to occur in the eye, sinuses, gastrointestinal tract, vagina, and uterus, in addition to the skin. We report these cases because we believe that our case report will be of interest to physicians who treat the above organs.

## Article Information

### Conflicts of Interest

None

### Author Contributions

Drs. Matsushita and Oishi had full access to all the data in this report and take responsibility for the integrity of the data and the accuracy of the data analysis.

Acquisition of data: Drs. Oishi, Fushida, Nishio, Fujii, Horii, Shimizu, Maeda, Hamaguchi, and Matsushita.

Drafting of the manuscript: Drs. Oishi, and Matsushita.

All authors read and approved of the final manuscript.

### Informed Consent

Written informed consent was obtained from the patient for the publication of this case report.
